# Living Lab for Citizens’ Wellness: A Case of Maintaining and Improving a Healthy Diet under the COVID-19 Pandemic

**DOI:** 10.3390/ijerph19031254

**Published:** 2022-01-23

**Authors:** Natsuko Tabata, Mai Tsukada, Kozue Kubo, Yuri Inoue, Reiko Miroku, Fumihiko Odashima, Koichiro Shiratori, Takashi Sekiya, Shintaro Sengoku, Hideaki Shiroyama, Hiromichi Kimura

**Affiliations:** 1 Institute for Future Initiatives, The University of Tokyo, Tokyo 113-0033, Japan; tabata@ifi.u-tokyo.ac.jp (N.T.); odashima@ifi.u-tokyo.ac.jp (F.O.); k.shiratori@ipu-japan.ac.jp (K.S.); sekiya@ifi.u-tokyo.ac.jp (T.S.); siroyama@j.u-tokyo.ac.jp (H.S.); hkimura@ifi.u-tokyo.ac.jp (H.K.); 2 Higashiyamato City, Tokyo 207-8585, Japan; ssengoku@ifi.u-tokyo.ac.jp (M.T.); k.kubo@iog.u-tokyo.ac.jp (K.K.); yuri.inoue@iog.u-tokyo.ac.jp (Y.I.); kenkou@city.higashiyamato.lg.jp (R.M.); 3 Institute of Gerontology, The University of Tokyo, Tokyo 113-0033, Japan; 4 School of Environment and Society, Tokyo Institute of Technology, Tokyo 108-0023, Japan; 5 Graduate School of Public Policy, The University of Tokyo, Tokyo 113-0033, Japan

**Keywords:** wellness, living lab, public health, local government, metabolomics, intestinal environment

## Abstract

The establishment and implementation of a healthy lifestyle is fundamental to public health and is an important issue for working-aged people, as it affects not only them but also the future generations. However, due to the COVID-19 pandemic and associated behavioural restrictions, lifestyles have altered, and, in certain environments, significantly worsened. In the present study, we conducted a project to improve the intestinal environment by focussing on the dietary habits of participants, utilising the living laboratory as a social technology to explore how to adapt to this drastic environmental change. We held eight workshops for voluntary participants and implemented a self-monitoring process of recording dietary behaviours (n = 78) and testing the intestinal environment (n = 14). Through this initiative, we developed a personalised wellness enhancement programme based on collaboration with multiple stakeholders and a framework for using personal data for research and practical purposes. These results provide an approach for promoting voluntary participation and behavioural changes among people, especially under the COVID-19 pandemic, as well as a practical basis for the government, academia, and industry to intervene effectively in raising people’s awareness of health and wellness.

## 1. Introduction

### 1.1. Research Background

Good health and longevity are extremely gratifying, but it is difficult to achieve and maintain wellness, including mental health and healthy social activities, throughout life. The significance of discovering and practising one’s desired lifestyle from an early stage of life is generally discussed from the perspectives of both the individual and society. However, it is arduous for many people to change their habits and lifestyles cultivated over time in a purpose-oriented manner, maintain the changes, and improve them sustainably. In particular, the recent COVID-19 pandemic has brought into question the nature of healthcare and wellness.

Lifestyle, including dietary habits and social activities, is an inevitable part of the debate on the response to the COVID-19 pandemic [1]. A nutritionally balanced diet may not only maintain good health in a general sense but may also reportedly replenish the host’s gut microbiota with beneficial microorganisms, conferring a range of health benefits on the host, including improved immunity [2]. There have been reports that lockdown has positively impacted on health, with greater consumption of traditional, healthy meals prepared at home, and improved lifestyle habits such as increased exercise time and smoking cessation [3]. On the other hand, obesity is one of the factors that increase the severity of illness and mortality after COVID-19 infection [4] and, in some cases, there is concern about weight gain and an increase in the obese population due to lockdown and activity restriction [5,6,7]. Beyond physical health, how we maintain mental health and mindfulness is also important [8]. While people’s group activities are the greatest risk factor for the spread of infection, it is known that the strength of social connections during COVID-19 lockdown is associated with less distress and fatigue in the public [9,10]; discussion should be devoted to resolving this paradox. A different set of social technologies and institutional responses are required to enable citizens to maintain healthy, infection-preventive lifestyles and the social and human connections that support mindfulness, and to respond flexibly to the COVID-19 pandemic situation [11].

### 1.2. Diet and the Intestinal Environment

In the present study, the intestinal environment test was used as an aid to health management since the gut microbiota is associated with a variety of risk factors for COVID-19 infection, and the optimisation of the gut environment is a potential therapeutic target for multiple diseases. In the human intestine, there are 1000 species and 40 trillion complex intestinal microflora, and metabolites produced by them are involved in the maintenance of human health and control of various diseases from the first year of life [12]. In particular, the intestinal environment during infancy is associated with future health risks, such as the development of childhood allergies [13,14,15]. Therefore, it is considered beneficial to properly understand the intestinal environment of infants in order to suggest and plan lifestyle and dietary habits for their healthy growth [16,17,18,19]. Moreover, the gastrointestinal tract is an important organ that influences the incidence and severity of COVID-19 infection [20,21].

Short-chain fatty acids are among the most important indicators of host health among metabolites [22]. They are metabolites of intestinal bacteria absorbed from the mucosal tissues of the colon, and used for the proliferation of epithelial cells, secretion of mucus, and as an energy source [23]. Additionally, some of them are transported to the whole body via bloodstreams and used as a material for synthesising fat in the liver, muscles, kidneys, and other tissues [24]. They also perform a variety of other functions, such as inhibiting the growth of harmful bacteria and stimulating the mucous membranes of the large intestine to promote peristalsis [23,24]. In particular, butyrate or butyric acid has been reported to induce the differentiation of regulatory T cells, which control the host’s immune system [24,25,26,27].

Especially in the case of infants, bifidobacteria predominate in the intestine from birth to before the start of weaning food, and bifidobacteria metabolise various sugars, including human milk oligosaccharides contained in breast milk, to produce short-chain fatty acids, such as lactic and acetic acids [22,23,28,29]. Therefore, when short-chain fatty acids are abundant in stool, it can be presumed that bifidobacteria with the ability to produce short-chain fatty acids are actively engaged in metabolic activities in the intestinal environment. An inverse relationship between the pH of the stool and the concentration of lactic acid in the stool of infants was observed [28,29,30]. Additionally, the pH of the stool was lower in infants with bacteria that metabolise human milk oligosaccharides in breast milk to produce acetic acid [28,29,31]—this measurement of stool can be used as an indicator of the amount of short-chain fatty acids in stool and the presence of bacteria that are considered beneficial.

### 1.3. Libing Lab

Living labs are social devices that have been used in many social innovation methods to solve complex social problems [32,33]; it is currently expected that these will help in the response to the COVID-19 pandemic through the further development of open innovation. Although open innovation, as proposed by Chesbrough [34], is a concept of new value creation in corporate strategy, it has developed independently in the context of social innovation and has evolved into an innovation style for the simultaneous pursuit of economic development and sustainability (Open Innovation 2.0) [35]. Today, living labs are considered a useful instrument for detecting communities’ needs, advancing local development, and contributing to innovation policies and local governance processes [36,37]. 

Currently, there are more than 400 active projects—and more under constant development—on the subjects of social acceptance of new initiatives and social systems design in the areas of mobility [38], energy sustainability [39] and healthcare [40], and the social implementation of new technologies such as healthcare and internet of things (IoT) [41,42]. In Japan, living labs have been implemented in various environments [43,44]. These include diverse attributes, such as leader (government, academia, business, etc.), regional characteristics (urban, rural, etc.), and facility characteristics (public facility, hospital, etc. [44]. At the same time, there is a need to balance economic a societal resilience and the resolution of various types of issues, including the COVID-19 pandemic [45]. Such complexity of the environment is increasing companies’ interest in living labs and facilitating transdisciplinary developments for problem solving [46].

### 1.4. Aim and Objectives

In light of the above context, the present study empirically set up and operated a living lab to put the model into practice and explore pathways for behavioural change and sustainable problem-solving in the context of industry-government-academia-private collaboration and innovation: the so-called Quadruple Helix Open Innovation model [47,48]. More specifically, the present study employed a demonstration field in Tokyo, Japan and collaborated with the city residents in a dedicated living lab. Thereafter, we conducted an exploratory lifestyle design project at the living lab to identify and develop healthier conditions by setting the following research questions: (1) what are the key factors for success (KFS) for effective and sustainable operation of a living lab?; (2) how to embed corporate innovation initiatives in the operation of a living lab?; and, in particular, (3) what is the significance and utility of a living lab in the context of the COVID-19 pandemic? Subsequently, we describe the series of initiatives and discuss the key points of the framework for collaboration among industry, government, academia, and the private sector, which serves as the basis for the initiatives, and examine the process and mechanism of the transformation of citizens’ lifestyles that result from the practices.

## 2. Materials and Methods

### 2.1. The Case

Higashiyamato City (hereinafter referred to as ‘the city’), located in the Tama region of the Tokyo Metropolis, has a population of about 85,000 and an area of 13.42 sq. km. It was a rural area, but, since around 1960, housing complexes have been built and population has dramatically increased. The city has been promoting measures to support children and child-rearing under the slogan of ‘creating the best city for child-rearing in Japan’. Collaboration among industry, government, academia, and the private sector began in earnest in May 2019, when the city and the Institute for Future Initiatives, the University of Tokyo (hereinafter ‘the research institute’) concluded a partnership agreement on citizens' health promotion. Based on this agreement, the Comfortable Intestines Project (hereinafter ‘the project’) was designed and implemented at the Higashiyamato Lifestyle Lab (hereinafter ‘the living lab’) in order to promote healthy dietary habits for infants and the child-rearing generation as a lifestyle design to promote citizens’ health.

### 2.2. Organisations and Collaborative Scheme

**Figure 1:**
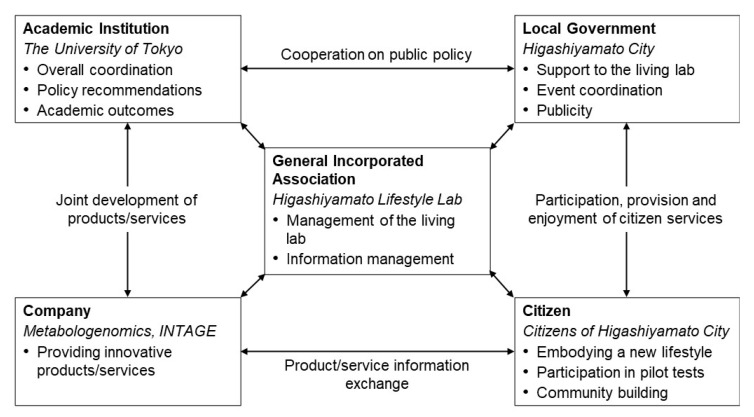
Implementation structure of this project. Bold indicates the sector involved; italics indicate the actors in this case.

To start the programme, we established the organisational structure shown in Figure 1. An incorporated association was established as the operating entity; the collected data were accumulated in accordance with the institutional review board’s regulations. In collaboration with several companies, INTAGE HOLDINGS Inc. (Chiyoda, Tokyo, Japan) provided operational funding and cooperated in data analysis and evaluation; Metabologenomics Inc. (Tsuruoka, Yamagata, Japan; hereinafter ‘the company’), a biotech company dedicated to metabolomics, cooperated in the provision of biological tests and expertise.

### 2.3. Project Design and Implementation

**Figure 2:**
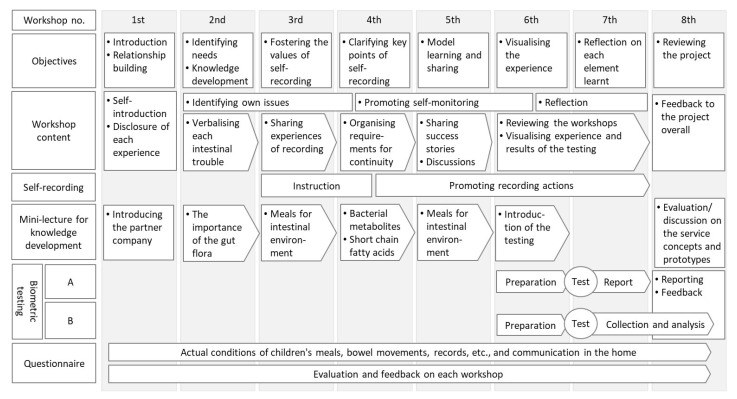
The process and steps of the programme. The self-recording refers to the process of recording the dietary habits of participants and their families by themselves. The bibliometric testing consisted of the existing intestinal microflora test (‘A’) and the short-chain fatty acid simple test (‘B’).

The programme process and itinerary are shown in Figure 2. This programme, implemented in 2020, comprised eight workshops. Each workshop comprised objectives, workshop content, self-recording, mini-lectures for knowledge development, biometric testing, and questionnaires. The venue was a community centre in the city, and staff from both the city administration and the research institute were involved in the operation. An overview of each workshop is provided in Appendix A.

### 2.4. Measurement and Analytical Methods

#### 2.4.1. Recruiting Test Subjects

Voluntary participants in the project were recruited by the health division of the city administration. Specifically, leaflets-based announcements were made to first-time mothers in childcare consultation meetings, visitors to the city health centre, and visitors to the city hall.

In order to ensure that the applicants to the workshop understood that the programme was an aspect of collaborative research between industry, academia and the public, the following information was provided in the application and consent form and their consent was obtained: the purpose and significance of the research; the methods of the research (inclusion/exclusion criteria, items to be included, implementation flow, testing flow, and handling of samples and data), the privacy policy, compliance, the storage and disposal policy of samples and data, the policy on secondary use of samples and data, the information disclosure policy, and confidentiality.

#### 2.4.2. Intervention Index

In this project, two types of tests were applied: the existing intestinal microflora test (‘test A’) [30], and the short-chain fatty acid simple test (‘test B’) [31], which is a simple test under development. Both tests were conducted by the company. In this project, the flow of the tests, the attitudes of the participants towards the tests, and their reactions to the results were investigated. The outline of the tests is as follows. The test A is a mass sequence analysis using a next-generation sequencer for PCR products that amplify the 16S rRNA region (MG Navi^®^). The test B focusses on short-chain fatty acids that are detectable using an analytical kit that can easily check and monitor the intestinal environment, which has been exclusively developed by the company.

#### 2.4.3. Testing Process

The inspection process is as follows.

- Subject: Registered participants of the 6th workshop and their children (total 14 groups: 14 adults and 17 children), including those who could not participate on the day of the workshop but wished to register later.
- Requirements for participation: Participation in the 7th and 8th workshops, cooperation in questionnaires, and sharing of test result data.
- Test contents: Intestinal microbiota test (test A, sample submission), short-chain fatty acid simple test (test B, image submission).
- Explanation and training procedure: The whole testing procedure was explained to the participants in the 6th workshop by a demonstration of the actual kits and a slide presentation. These kits are designed for general consumer use and, thus, do not require any special skill or expertise.
- Distribution of the tools: The test kit was distributed in the 6th workshop after collecting the application and consent form from those who wished to participate. Those who wished to participate but were absent were individually explained at a later date in the same way.

#### 2.4.4. Testing Flow: Test A (Intestinal Microbiota Test)

- The participants collected their child’s stool from diapers or wipes at home in a revalved container (MG Kit) and sent it to the company in a special envelope. All the storage and transport processes were carried out at room temperature.
- The results were returned to the participants individually in a paper form in the 8th workshop. The sample itself was disposed of by the testing company after the analysis in accordance with the defined regulations.
- The moderator of the company gave a lecture on the overall trend, followed by discussions and Q&A, in the same workshop. The detail of the relevant workshop was described in Appendix A.

#### 2.4.5. Test Flow: Test B (Short-Chain Fatty Acid Test)

- The participants prepared image samples at home through the following procedure: (i) Collected stool from diaper using swab; (ii) suspended stool in the designated container, attached the cap with nozzle, and dripped the stool suspension onto the sensor through the nozzle; (iii) read and recorded the discoloration of the sensor; (iv) took a picture of the sensor, and (v) sent to the specified destination via a Google Form. The sample itself was disposed of by the participant themselves as a normal waste.
- The results were returned to the participants individually in a paper form in the 7th workshop.
- The moderator of the company gave a lecture on the overall trend, followed by discussion and Q&A, in the same workshop. The detail of the relevant workshop was described in Appendix A.

#### 2.4.6. Questionnaire Survey

The contents and results of the questionnaire surveys are presented in Appendix B. A Google Form was used to complete the questionnaire.

## 3. Results

### 3.1. Implementation Theme and Approaches

To set the implementation theme, we collected information via group interviews conducted at childcare consultation meetings. These interviews were based on the results of questionnaire surveys conducted by the city administration in the past (data not shown). Throughout these investigations, we finally adopted intestinal health as the theme and named the project the ‘Comfortable Intestines Project’, because participants discussed the amount of vegetables they consumed and exhibited a high level of interest in improving their intestinal environment.

The target population was set as productive generation. Additionally, the city administration had been taking active measures to support child rearing, so we decided to first target the parents of preschool children who are highly interested in health. In designing the implementation process, we discussed among the stakeholders, as shown in Figure 1 and Figure 2. This being the inaugural year, the project was designed as a hypothesis-testing project, and the activities of the living laboratory were implemented.

### 3.2. Workshops

**Figure 3:**
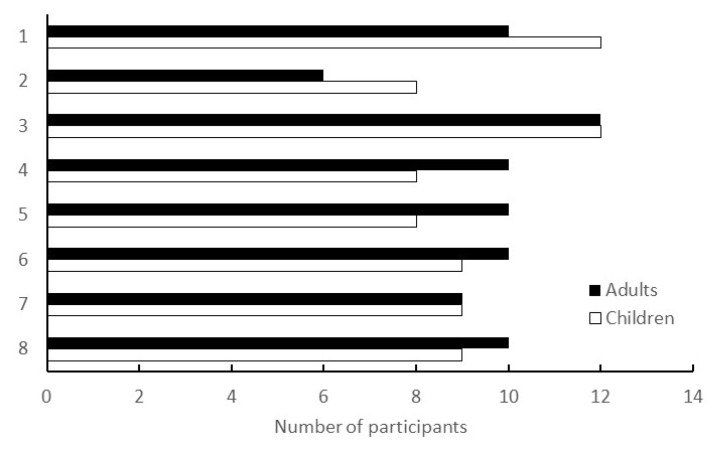
Number of participants in the workshop. The vertical axis represents the number of times the workshop was conducted; the horizontal axis represents the number of participants at each workshop. Children were dependents of adults and did not participate in the workshops; they were left at the attached childcare facility.

Workshops were conducted in a face-to-face format at the living lab. Based on previous research and past findings of the city, the workshops were designed to be held periodically—about once a month—and were held eight times, from June 2020 to March 2021. The number of participants in each session is shown in Figure 3.

**Figure 4:**
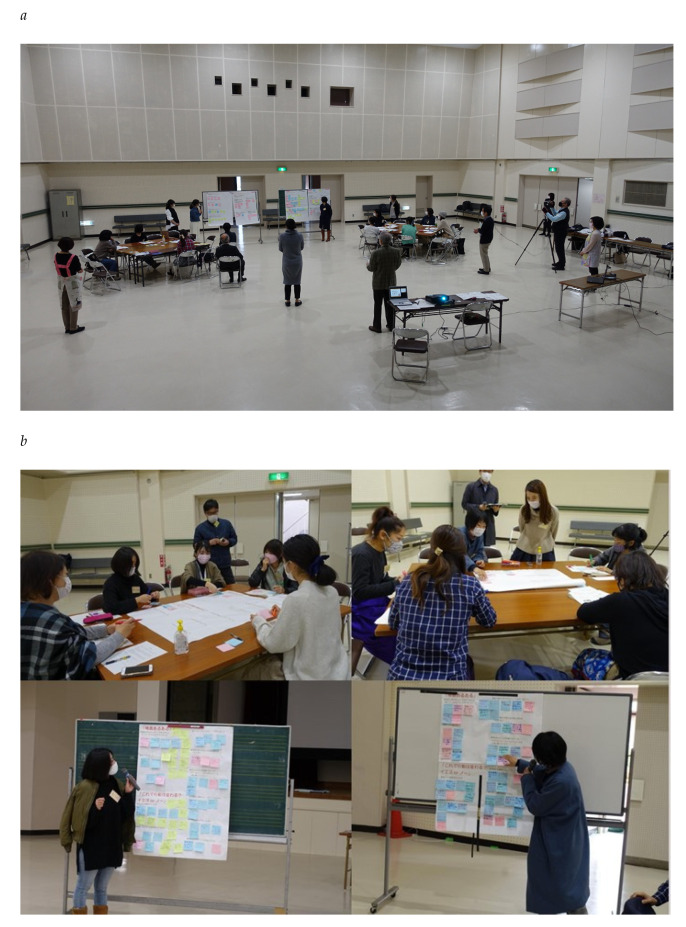
Scene of the workshop venue. The following is an example of the discussion process. (**a**), General view of the 8th workshop. (**b**), The discussion process during the 7th workshop. Participants were divided into two groups (left and right) and desk-based discussions (top) and board-based presentations (bottom) were conducted.

As the workshops were held during the COVID-19 pandemic, the first priority was to ensure adherence to COVID-appropriate behaviour. Thus, to ensure social distancing, we reduced the number of participants. The workshops were held in the community centre hall operated by the city administration to avoid intensive contacts for both participants and management staff; efforts were also made to ensure proper ventilation (Figure 4). Participants were required to measure their temperature, disinfect their hands, and check their physical condition upon arrival. During the workshops, no one complained of ill health at the time of arrival or after returning home.

### 3.3. Outcomes

**Figure 5:**
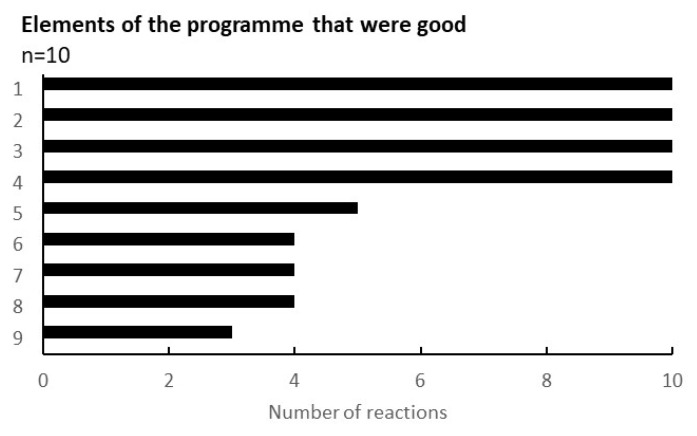
Elements of the programme that were good. The results were taken from the survey conducted during the eighth workshop. The vertical elements were as follows: 1, I could have my child taken care of safely; 2, I could have a health examination; 3, I could learn about the intestinal environment; 4, I could listen to others in a similar situation; 5, the workshop was held in my neighbourhood and it was easy to attend; 6, I could relate to the concept of ‘health & wealth’; 7, the workshop method was interesting; 8, They will listen to my stories and worries; 9, I could reconnect with previous participants.

One of the outcomes obtained through the programme was the feedback from the participating citizens in the workshop. Figure 5 shows the results regarding the evaluation points of the workshop. Out of 9 questions, 4 points were agreed by all recipients (n = 10): the environment of the workshop (childcare specifically), the opportunity for the health examination, the better understanding of the gut environment, and the dialogue with others.

In addition, the participants provided feedback on the intervention methods. According to the results of the questionnaire on the operation of the device for the Test A, 16 out of 17 respondents answered that it was ‘easier than expected’, and the three sample submissions were made without delay, confirming that the self-test per se is not difficult for the general public. However, the process of reading and recording the discoloration of the sensor in the Test B was evaluated as ‘difficult’ by 6 out of 17 respondents in the first submission, and by 5 out of 16 respondents in the second submission. 

The second outcome is a trial to improve the product used in the intervention (test B), based on the feedback described above. A comparison of the readings by the user and the researcher, based on the submitted images, revealed a gap in colouring by exposure in approximately half the cases (data not shown). Additionally, some of the participants’ image data sent to the company were processed using photo applications, making it difficult to perform accurate analysis. To solve these problems, the necessity of developing an application that reads the sensor on the spot and displays appropriate numerical values was recognised. Additionally, the disposal method of the mount containing the sensor, the disposal method of the container and cotton swab used to prepare the stool suspension, the time from defaecation to inspection, and the time from dropping to reading were identified as issues that needed to be reviewed, leading to a review of the included items and the contents of the instructions.

## 4. Discussion

### 4.1. Features of the Case Study

**Figure 6:**
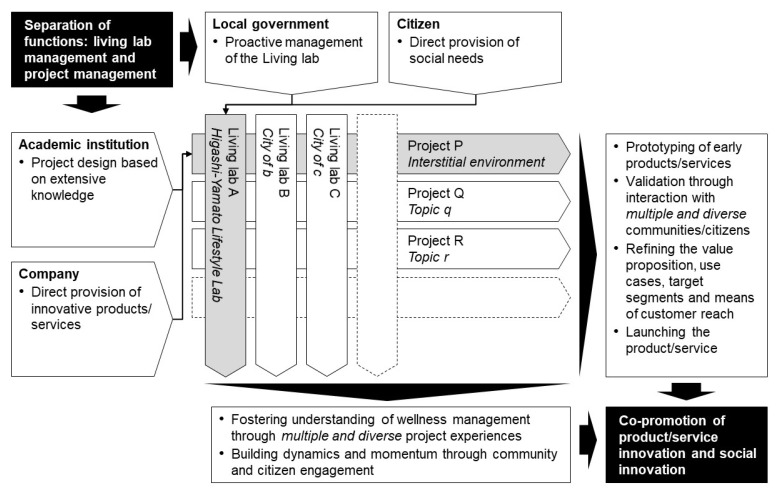
Programme design consisting of multiple projects and living labs. Shaded areas indicate the present case, and blank areas indicate generalised ones.

The programme design comprises one of the features. While initiatives for the use of living labs have started in various places—mostly based on high expectations—there is no clear and unified definition, and, in Japan, in particular, knowledge has not been systematically organised [49]. Additionally, there is a concern that even if a long-term vision is shared, it may not meet short-term expectations because of power issues among stakeholders and the reluctance of end-users, given the wide range of stakeholders [50]. Therefore, in this study, the functions of the project and the living lab were intentionally separated in the design of the programme, and each was defined and agreed upon. By doing so, the position and role of each stakeholder were clarified, and a good collaborative (and tense) relationship was fostered between them. As a future perspective, the formation of a ‘market’ for multiple projects and multiple living labs can achieve both scalability of a project, i.e., it can be deployed in various regions and environments, and continuity of a living lab, i.e., appropriate projects can be selected and implemented at any time. Figure 6 shows a schematic of this concept.

The second feature is the introduction of the self-monitoring method of listening, recording, and sample testing. It is one of the important functions of the living lab to be able to elicit honest and frank opinions of citizens. The primary goal of each workshop was to deepen the participants’ understanding of themselves and elicit their own output. In health psychology, self-monitoring is considered to contribute the most to the effectiveness of interventions in the process of behavioural change, regardless of the health behaviours that are focussed on [51]. In the present programme, participants who were able to record their bowel movements informed that their health improved because they became more aware of their bowel rhythm by recording them, and that their awareness of food choices improved. In other words, intestinal health became the ‘theme of health improvement’ and the recording act became the ‘health improvement behaviour’. Additionally, the health belief model of behavioural science [52] was applied and realised.

Finally, cutting-edge technology and innovative elements were implemented. In this study, the participation of an academic institution with advanced knowledge, and a start-up company that develops and provides new products and services enabled us to provide participants with content that could not be provided by the local government alone. Similarly, for these stakeholders, participation in this research was an opportunity for social system design research and test marketing of new products and services. Consequently, a mutually beneficial relationship was established. This point will be discussed in detail in the following section.

### 4.2. Significance and Utility under the COVID-19 Pandemic

The COVID-19 pandemic spread in Japan immediately after the briefing session in February 2020, when this study was initiated. While many public projects were cancelled or postponed because of the declaration of a state of emergency, all eight workshops of the programme were physically held as scheduled, with a total of 78 participants, 6–12 for each session. The following points suggest why we were able to conduct the programme in such a difficult environment, and its significance and benefits.

#### 4.2.1. Appeal to the Significance of Health Promotion under the COVID-19 Pandemic

The mission of the city government to promote the health of its residents had been widespread, and under this mission, the living lab was promoted and deeply understood by stakeholders as an important opportunity to improve lifestyles and maintain health. Accordingly, the framework for implementation was managed flexibly according to the situation. COVID-19 was a major concern; however, at the same time, the reduction in the level of physical activity due to home isolation, and the loss of communication and stress reduction opportunities due to the severe restriction of social activities caused serious health problems, especially psychological problems, which resulted in many suicides. Thus, the successful conducting of the programme can be attributed to comprehensive health promotion and risk management under the aforementioned mission of the city government.

#### 4.2.2. Local Government-Led Programme Coordination

According to previous research, successful living labs often leverage existing communities [53]. The city is a direct municipality for citizens, and one of the success factors may be the fact that the city established a framework to collaborate with various stakeholders in an attitude-driven manner, implement programmes for citizens, and reuse the data for research. Specifically, the project established an infrastructure for organising contracts and information, acquiring and retaining personal and laboratory information of participating citizens, and establishing a cooperative framework for project promotion, mainly in the city. In terms of recruiting participants, the city’s health department played an active role in announcing the project at events, securing a safe location, planning the programme, and mentoring and facilitating participants in the workshops.

#### 4.2.3. Adapting to the Potential Needs of Citizens

Active involvement and proactive efforts of the participants were important factors. Even though it is an important lifestyle element, it was a concern when we first discussed with the idea whether participants would be able to share the sensitive topic of their own and their children’s defaecation with others and whether we would be able to encourage their active participation. However, the results showed participants’ active engagement at each workshop, and satisfaction with participation, willingness to participate, and willingness to be referred were given high ratings by them. In free comments, they shared thoughts such as the following: ‘It was good to be able to talk about things that are difficult to talk about’ (such as stools and bowel movements), and ‘It is good to be able to listen to other people’s stories’ (see Figure 5). This adaptation to the participants’ needs is probably the result of careful verification of past measures and the preliminary survey using group interviews, as explained in Section 2.

#### 4.2.4. Optimised Environment Development for Participants

In terms of communication during the workshop, we used nicknames for everyone, including the staff (so that they would not know each other’s personal information), established rules for discussions and group work, appointed facilitators (so that the participants would not become aggressive), and allocated time for ice-breaking (not only for the participants, but for everyone in the hall, including the staff, to introduce themselves for the episode). Additionally, the method of voting with stickers was adopted.

The provision of temporary childcare during the workshops was particularly appreciated by the participants (see Figure 5). Based on our experience at the briefing session of the living lab, where children continuously moved about the room, negating parents’ calm participation, a temporary childcare facility in a separate room was set up during all the workshops. This is the main reason for the extremely high participation rates of mothers and children—despite the COVID-19 pandemic.

### 4.3. Significance and Utility of Technology and Innovative Elements

This study also provided useful insights into this service for the company, which designed and provided the test (Dr Taku Nakahara of Metabologenomics, Inc., personal communication). This workshop was also an opportunity to validate a new testing methodology, specifically, the short-chain fatty acid simple test (test B). Although there was an inducement factor of feedback of the test results for the participants, there was a concern that they would resist an unestablished test. Consequently, despite it being a first-time test, workshop participants actively participated in it (100%, n = 14), achieved an extremely high sample recovery rate (94.1%, n = 17), and many of them reported not feeling any psychological resistance. Following active discussions, diverse opinions were collected during the workshop (data not shown). As for the value of this test, some opinions led to specific actions, such as ‘it will be a way to be careful in daily life based on the results,’ (Appendix B); it was estimated that the means of providing information and the selection of appropriate timing contributed to the level of satisfaction. This could be due to the fact that trust in the city government and the place of implementation had been fostered, and the flow from the examination to the understanding of physical condition was smooth.

### 4.4. Limitations and Prospects

Finally, we discuss the limitations of this study. First, the limitation of the number of participants due to a declaration of state of emergency, and the limitation of the research design that could not include a non-intervention group due to the government’s commitment to equal participation; statistical analysis based on measured data and survey results could not be performed for this initiative; it was limited to an approach theory of the health theme and its promotion methods. It is necessary to iteratively implement this theme and methodology in a future project at a living lab and analyse it based on large-scale data. Second, although biometric testing met the need of participants to know their condition, it was not easy to meet the strong need for causal information. In the future, it will be necessary to analyse causal relationships by utilising the results of known research and improving the accuracy of simple tests. Third, although the satisfaction level of participants was extremely high, the scale of implementation had to be reduced because of the COVID-19 pandemic. In addition to the repetitive implementation described above, raising awareness regarding the significance and utility of the programme and its horizontal expansion to other municipalities are required.

## 5. Conclusions

In the present study, we focussed on dietary habits as a part of citizens’ health management during the COVID-19 pandemic and implemented a gut-health improvement project at a living lab. Consequently, we developed a wellness improvement programme for individuals based on the collaboration among various stakeholders, including local governments, academia, businesses, and citizens, and established a framework for reusing the acquired data for research and practice. The results showed that, for the three research questions: (1) the KFS were the programme design based on both the project and the living lab, the introduction of self-monitoring methods, the formation of context in the utilisation of lifelog data and the introduction of technology and innovative elements; (2) in particular, we confirmed that innovative approaches and services provided by the research university and the company were directly related to participants’ willingness to participate and high satisfaction; finally, (3) even during (or because of) the COVID-19 pandemic, the opportunity to learn about and discuss gut health and other health issues, and to listen to and share the stories of others in the same region and in similar circumstances, was highly valued. This case study confirmed the significance and benefits of wellness management led by local governments and matched to citizens’ needs, especially under the COVID-19 pandemic. Additionally, insights into how to create an optimised environment for participants were obtained.

## Figures and Tables

**Figure A1:**
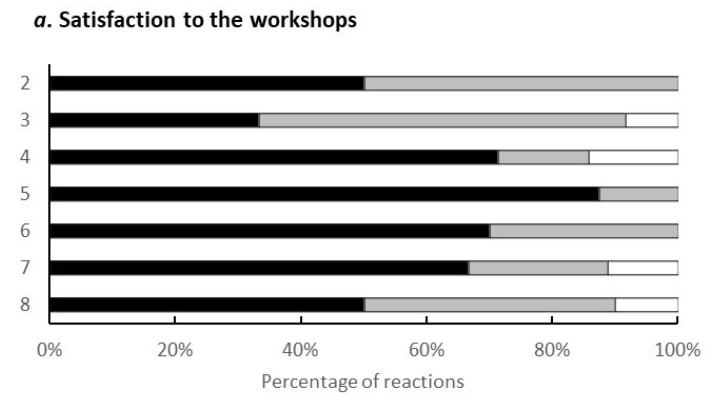

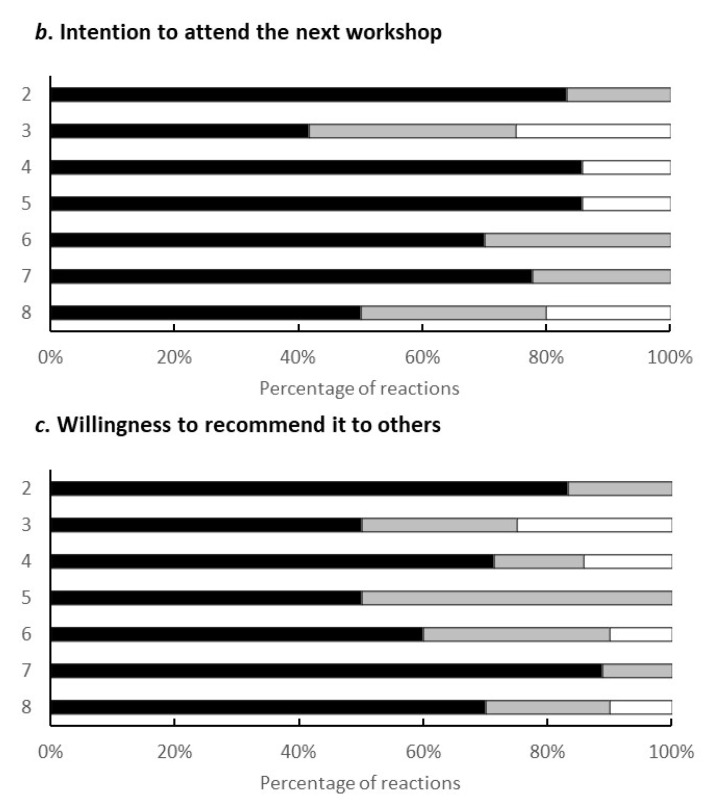
The vertical axis represents the number of times the workshop was held, and the horizontal axis represents the breakdown of respondents for each workshop. Black, grey and white bars exhibit ‘strongly agree’, ‘agree’ and ‘neither’, respectively.

## Appendix A. The Contents of the Workshops

1st Workshop (24 June 2020)

- Introduction and explanation of the main idea
  ○ Explanation of the purpose of the project
    ▪ Higashiyamato City’s approach
    ▪ What is a Living lab?
    ▪ Background of the theme, etc.
  ○ Discussed concerns during the COVID-19 self-restraint period
- Presentation on participants’ concerns

2nd Workshop (29 July 2020)

- Tummy Health Needs Deep Dive
  ○ Case work
    ▪ Write down the theme of ‘tummy troubles’ on a sticky note and present it to the group
    ▪ Name a tummy problem that you use as a reference
  ○ Presentation by 2 groups
    ▪ Affixing the empathy seal

3rd Workshop (20 August 2020)

- Digging deeper into the solution image
  ○ Review of last time
    ▪ Do you keep any ‘records’? What’s holding you from keeping a record? Filling in stickies and presentations in the group
    ▪ First impressions of existing tools: ‘Looks good’ and ‘Doesn’t look good’ on sticky notes and presentation in the group
    ▪ Fill-in the sticky notes of ‘My impression ranking’ and announce it to the group
  ○ Presentation by 2 groups
    ▪ Affixing the empathy seal
  ○ Homework for participants
    ▪ Let us try it before the next time: ‘Record Challenge (declared)!’
  ○ Intestinal Environment Mini Lecture

4th Workshop (23 September 2020)

- Experiences and deep dives
  ○ Review of last time
    ▪ For the recording method you chose, write on sticky notes: (i) How long did you keep the record, (ii) What was good or bad about the method, and (iii) Good/noticeable points about the record?
    ▪ Thinking with keyword cards, ‘If I do it this way, the record will last.’
  ○ Card game
    ▪ Filling in stickies and presentation in the group
  ○ Presentation by 2 groups
    ▪ Affixing the empathy seal
  ○ ‘This is the way I’d like to record it!’ Declaration
  ○ Intestinal Environment Mini Lecture

5th Workshop (28 October 2020)

- Record awareness search and deep investigation of gut-environment needs
  ○ Case work 1: ‘Hear your stories about how you have used the records.’
    ▪ Presentation by three participants who are recording well
    ▪ Write your findings and opinions on sticky notes and attach them to the graphic recording
  ○ Case work 2: ‘Keyword Card: Children’s Tummy Trouble’
    ▪ While discussing with the group whether or not they have had any experience with the contents of the ‘Tummy Trouble Card’ and their opinions, fill-in the sticky notes and attach them to a piece of paper.
  ○ Intestinal Environment Mini Lecture

6th Workshop (25 November 2020)

- Explanation of the Intestinal Environment Test and creation of the Customer Journey Map
  ○ Explanation of the Intestinal Environment Test
    ▪ pH test, bacterial flora test, and testing system for children’s stool
    ▪ Fill-out the test consent form
    ▪ Test kit distribution
  ○ Case work
    ▪ ‘Visualising Your Experience in the Lifestyle Lab.’
    ▪ Individual work: filling-out the My Experience Summary Sheet
    ▪ Group work: ‘Let’s summarise everyone’s experiences into one story!’

7th Workshop (27 January 2021)

- Sharing of simple test results and opinions, sharing of experiences
  ○ Results of the Intestinal Environment Test B (short chain-fatty acid)
    ▪ Introduction of opinions that lead to improvement
    ▪ What are high levels of short-chain fatty acids associated with?
    ▪ Results summary
  ○ Case work
    ▪ Will this change your behaviour? Yes or no.
  ○ Presentation by group representatives

8th Workshop (10 March 2021)

- Sharing the results of the intestinal microbiota test, sharing opinions, and reflecting on the results
  ○ Results of the Intestinal Environment Test A (intestinal microbiota test)
    ▪ ‘How to read the test results?’
  ○ Case work
    ▪ ‘Reading through the test results.’
    ▪ ‘Did your participation in the lab change anything? Has it helped you?’
  ○ Presentation by group representatives

## Appendix B. Questionnaire Survey Results

Evaluation Results for the Programme (Figure A1).

Free answers

Q. I would like to ask you about the intestinal environment test. Please tell us why you would like, or not like, to take the test. Questionnaire in the seventh workshop; all respondents answered that they would like to take the test.

- Because my child tends to be constipated.
- It’s nice to be able to look it up because even though I am a little worried, to this day I still do not know if my kids’ poop is good or bad.
- Because I was always worried about my son’s intestinal environment (constipation since he was 0 years old)

Q. Please tell us about your reasons, background, and expectations when you first decided to participate in this lab. (Questionnaire in the eighth workshop)

- I wanted to have a chance to think about my child’s health. They can take care of my children.
- The reason I decided to participate was to learn about stool.
- I was worried at first because I had to take care of my child at the same time, but I was able to do it without any problems.
- I was approached by the people involved and it came with childcare.

Q. Finally, if you have any requests or messages about the management of the association, please feel free to write anything. (Questionnaire in the eighth workshop)

- I am sure you have faced enormous trouble holding these meetings during the COVID-19 pandemic, and I thank you for making it possible for us to attend each meeting with peace of mind. Participating in these meetings, even if only once a month, is a good refresher for me and my son, and it has given us a chance to seriously consider food and the intestinal environment, which we have never done before. I am glad that I have participated. Thank you very much.
- I really enjoyed participating in each session. I learned a lot, did the tests, and my child seemed to enjoy playing with the nursery staff and friends. The preparations in advance were meticulous, and we spent a lot of time together. Thank you very much.
